# Molecular Markers Improve Abiotic Stress Tolerance in Crops: A Review

**DOI:** 10.3390/plants9101374

**Published:** 2020-10-15

**Authors:** Adnan Younis, Fahad Ramzan, Yasir Ramzan, Faisal Zulfiqar, Muhammad Ahsan, Ki Byung Lim

**Affiliations:** 1 Institute of Horticultural Sciences, University of Agriculture, Faisalabad 38040, Pakistan; adnanyounis@uaf.edu.pk (A.Y.); ch.faisal.zulfiqar@gmail.com (F.Z.); 2 Department of Horticulture, Kyungpook National University, Daegu 41566, Korea; fahidflorist@yahoo.com; 3 Wheat Research Institute, Ayub Agricultural Research Institute, Faisalabad 38850, Pakistan; yasiragrii@gmail.com; 4 Department of Horticultural Sciences, Faculty of Agriculture & Environmental Sciences, The Islamia University of Bahawalpur, Punjab 63100, Pakistan; ahsan.horti@iub.edu.pk

**Keywords:** DNA, genotype, salinity, drought, heat

## Abstract

Plants endure many abiotic stresses, such as temperature (heat or frost), drought, and salt. Such factors are primary and frequent stressors that reduce agriculture crop yields. Often alterations in nutrient management and constituents, along with variations in biosynthetic capacity, ultimately reduce or halt plant growth. Genetically, stress is an environmental condition that interferes with complete genetic expression. A vast range of molecular genomic markers is available for the analysis of agricultural crops. These markers are classified into various groups based on how the markers are used: RAPD (Random amplified polymorphic DNA) markers serve to identify and screen hybrids based on salinity and drought stress tolerance, while simple sequence repeat (SSR) markers are excellent for the assessment of stress tolerance. Such markers also play an important role in the QTL (Quantitative trait loci) mapping of stress-related genes. Dehydrins for drought and saltol for salinity stresses are primitive genes which regulate responses to these conditions. Further, a focus on traits using single-gene single nucleotide polymorphisms (SNP) markers supports genetic mapping and the sequencing of stress-related traits in inbred lines. DNA markers facilitate marker-assisted breeding to enhance abiotic stress tolerance using advanced techniques and marker modification.

## 1. Introduction

Plants endure various unfavorable climatic conditions during their growth cycles. Such conditions are comprised of biotic stresses, including attack by herbivores and infection by pathogens, and abiotic stresses, including heat and cold, drought, scarcity of nutrients, higher levels of salt, and hazardous metals and metalloids (arsenic, cadmium, and aluminum) in the soil. Temperature (heat or frost), drought, and salt are the primary and most frequently encountered climatic factors that reduce agricultural crop yields. Such impacts are a dangerous sign for food security and also impact the geographical distribution of plants in nature. Climate change, i.e., long-term changes in weather patterns, is a source of significant abiotic stress [1,2]. The constitutive basal defense systems of plants are triggered after the recognition of stress. Multiple signaling pathways are differentially activated depending on the type of stress. Typical defense pathways are regulated by kinase enzymes and phytohormones. For example, ion channels are stimulated by jasmonic acid, abscisic acid, ethylene, and salicylic acid and through the generation of reactive oxygen species (ROS). These factors accumulate and reprogram genetic and metabolic machinery. Defense responses reduce the biological loss caused by stress; these processes are the basis of plant tolerance [3].

Crop production and yield are mainly affected by abiotic stresses because of adverse changes in environmental conditions [4]. An external factor that adversely affects plant growth or condition is usually labeled as a stress in biological terms [5]. Stresses are recognized as major diversions from normal plant life cycles. Stress-affected plants display three basic response phases: first is the alarm phase (initiation of stress), the second is the resistance phase (activation of defense systems), and the third is the exhaustion phase (loss due to stress) [6]. The effect of stress on the plant system is observed in many plants, affecting the growth of the plant [7,8,9,10]. Salinity is the primary climatic factor that restricts growth and productivity. It affects biological characteristics by promoting water acquisition and retention and altering ion homeostasis management [11]. Further, drought stress (regular scarcity of water) will cause the reduced survival, development, and growth of plants. Drought is usually associated with a lack of availability of water in the soil but can also be exacerbated by excessive evapotranspiration [12]. Such stress may occur under humid conditions and with high air temperatures, i.e., a higher temperature in the surrounding atmosphere. This stress is due to an imbalance between the water uptake from soil and water loss through evapotranspiration flux [13].

Genetically, stress is an environmental condition that stops a plant from obtaining complete genetic expression. Any stress not due to interactions with other organisms and that produces an adverse impact on organisms in an environment is considered to be an abiotic stress. Environmental and agricultural sector abiotic stresses are severe threats that are currently intensified by industrialization and global warming [14].

DNA markers have broad applications for improving a plant’s genetic structure, such as the genetic identification of parents, the assessment of variation on a genetic level, and the identification, genetic confirmation, and development of genetic linkage groups with high resolution. A vast range of molecular markers is available for the genetic analysis of crops. These markers are classified on the basis of how markers are utilized; e.g., PCR (Polymerase chain reaction)-based vs. non-PCR-based. DNA markers based on hybridization techniques are categorized as RFLP (Restriction fragment length polymorphism) markers. These markers were used extensively during the 20th century for the mapping of genes and in other genetic analysis approaches in the field of molecular biology. PCR was pioneered by Mullis and Faloona [15]. Their results powered advances in DNA marker systems and their utilization in genomic research. The time and expense needed for genetic mapping using probe hybridization was significantly reduced using PCR-based genetic markers. PCR is an in vitro technique that amplifies DNA sequences for a gene or locus. Primers are small oligonucleotides. In a target sequence, primers are complementary to adjacent gene sequences at both points of the sequence. The repeated cycling of DNA replication and melting produces large amounts of sequences of interest beginning from a small quantity of a single pattern [16]. Single nucleotide polymorphisms (SNP), sequence-characterized amplified regions (SCAR), and simple sequence repeats (SSR) are PCR-based molecular markers, and gene sequence information of the sample is required to use these molecular markers [16].

Molecular markers provide details with respect to the allelic position (heterozygosis, maternal homozygosis, paternal homozygosis) of every progeny or line in the population. Such inheritance structures can be checked and recorded. The development of linkage maps (genetic structure) usually includes the assembly of markers in a pattern that reflects genetic diversity and linkage assemblies based upon a recombination assessed by genotypes of hybrid plants [16,17]. Therefore, the objective of this review is to describe the impacts of abiotic stress on agricultural crops and associated DNA markers for genetic control, gene mapping, and the screening of stress resistance traits.

## 2. Abiotic Stress Impact on Agricultural Crops

Among abiotic stresses, heat and drought are the two major stresses that adversely affect the yield and productivity of a crop. Such abiotic stresses reduce farm income and agricultural benefits. The reduction of water by up to 40% causes the lowering of maize yields to 40% of the former yield and wheat to 21% of the former yield [18]. In Africa, an important agricultural crop, cowpea, currently faces drought stress, reducing yields by 34% to 68% [19]. Some abiotic stresses promote the excessive production of reactive oxygen species (ROS). ROS are toxic and reactive and destroy or damage carbohydrates, lipids, nucleic acids, and proteins. This oxidative stress adversely affects plant growth [20]. Further, water deficiency and heat stress can damage transpiration and stomatal conductance in plant leaves [21].

Agriculture production is affected by abiotic stresses. Within the world agriculture area, 91 percent of that area is under stress, and 50% of agriculture production loss is due to such stresses. The strength and harmful effects of abiotic stress may be accelerated with changes in climate. Improvement in agronomic management and stress-resistant genotype promotion in breeding programs can reduce this impact [22]. Abiotic stress has an impact on biochemical and physiological processes of plants. Improvement in the efficiency of light use and photosynthetic activity can increase tolerance against abiotic stresses. Furthermore, many antioxidants are activated, and various enzymes can develop stress-based metabolites to help in protection from cellular damage. However, there is a need to develop key adaptation strategies for increasing stress tolerance in plants [23].

Heat stress is closely linked to temperature. Increases in air and soil temperatures above tolerance levels for even short durations can affect crop development and growth [24]. Globally, an increase in temperature is a major climatic issue that may adversely affect production and growth of plants, specifically of crops. Increases in temperature will severely increase crop vulnerability. The study of heat stress is thus essential for understanding responses and tolerance of plants to such stress conditions. Ultimately, production and growth of progeny (lines) with greater heat tolerance will be necessary to sustain agriculture. Heat stress causes decreases in the germination of seeds, photosynthetic activity, and crop yields. Stages of reproductive cycles, and the function and roles of tapetal cells are reduced or stopped when flower anthers are dysplastic under heat stress. Higher temperatures may also stop pollen grain swelling and lead to the release of pollen with poor vigor. Anthers may also become indehiscence. Plant crops may need physiological or molecular modification to adequately respond to heat stress [25].

Many physiological changes are observed during drought stress, e.g., reduction in photosynthetic activity, variations in the elasticity of cell walls, and the closing of stomata. Notably, drought and salinity are related in their impacts on plant physiology and ultimately overlap within tolerance systems. Drought affects the nutritional status of crops by changing concentration levels of ions in plant tissues. Diffusion of available soil nutrients to root surfaces decreases along with moisture levels [26,27].

Alterations in nutrient constituents and management, along with variations in biosynthetic capacity, are main factors that ultimately reduce or halt plant growth. Protective systems for plant survival under abiotic stress are essential for maintaining crop growth and production levels in agricultural sectors. Abiotic stress defenses can be explored and understood using molecular genetics. Stress defense systems have been well studied with such methods, with a focus on stress tolerance [28].

Salinity, drought, heat, scarcity of nutrients, heavy metal levels, water/air pollution, light photoperiodicity, and intensity can all induce abiotic stress. These factors can affect plants individually or together and might ultimately alter the metabolic systems to reduce the productivity, development, and growth levels. Higher levels of stress may prove intolerable and result in plant death. Freedom from stress is not possible. Therefore, plants exhibit metabolic responses and specific molecules to survive under stressful environment [29,30].

Abiotic stress may necessitate changes in constituents and conditions of soil and plant environments that could lead to decreased yields of primary agricultural crops worldwide. Currently, agricultural lands in non-stressed regions comprise only 10% of crop production. The remaining 90% is facing one or more environmental stresses. Plants continue to adapt to abiotic stress biochemically, physiologically, molecularly, and phenotypically. Still, a persistent need exists for additional efforts to improve stress tolerance by improving plant defenses genetically, promoting technologies for resource conservation, and adopting other approaches [14].

## 3. DNA Marker Applications for Abiotic Stress Tolerance

### 3.1. RAPD Marker Analysis for Salinity and Drought Stresses

Random amplified polymorphic DNAs (RAPDs) are PCR-based markers. Preliminary sequence data of samples are not necessary in RAPD analysis. Many loci from many individuals can be analyzed for screening purposes using limited resources. RAPDs are widely used due to their easy experimental methodology and excellent genetic screening of intra- and interspecific hybrids [31].

These markers have been useful for identifying salinity stress tolerant genes in many crops. Various mechanisms are available to plants for tolerance/defense against salinity stress. Such responses are regulated genetically. Therefore, improving salinity tolerance in agricultural crops is critical, especially in saline-affected areas. DNA markers can help to identify and categorize salt-resistant genotypes. Utilizing PCR for RAPD amplification of specific DNA sequences is a basic approach for detecting salt-resistant genes. A study conducted in wheat to evaluate the genetic diversity of salt-resistant genotypes used plants growing in a saline-affected field. These DNA markers effectively differentiated salt-resistant from salt-sensitive genotypes. Polymorphic primer pairing between tolerant and sensitive genotypes confirmed genetic variation. Resistant wheat varieties might be developed using such genetic information to appropriately cross salt-resistant and salt-susceptible genotypes [32].

Various changes in DNA can be caused by salinity stress, such as structural breakdown and rearrangement. Such changes are due to secondary stress, e.g., oxidative destruction connected to the formation of ROS (hydroxyl radicals, singlet oxygen, superoxide, and hydrogen peroxide). RAPD markers help identify genetic instability of saline-affected cotton seedlings (NaCl treated). RAPD markers showed missing DNA band on agarose gels, weak or strong band intensity, and presence of new bands compared to control plant DNA. Previous findings confirmed that application of RAPD successfully investigated toxicological stress. OPA08 RAPD primer was informative and had significant potential for identifying DNA variations influenced by NaCl (saline) stress. Unfortunately, several issues still exist for the application of the RAPD technique. These issues include aspects of amplification and electrophoretic separation, such as contamination of DNA, identical band appearance, and competition in DNA amplification [33].

Field evaluation is comparatively laborious and time consuming for classifying quality parameters, stress resistance, and crop productivity. Molecular differences at the DNA level were successfully categorized in plants developed from tissue culturing [34]. Genetic screening of plants in stressful conditions has been performed by DNA marker technology [35]. RAPD is rapid and simple and requires only small amounts of DNA. The technique is sensitive to genetic differences and can quickly process large numbers of genomic samples under in vitro conditions. For example, polymorphic bands in maize representing salt-resistant genes were observed by RAPDs. Several primers were linked to salt resistance sequences that provided valuable information for breeding salt-resistant progeny. These progenies can be further screened by marker-assisted selection and developed for salt resistance by direct genetic modification of genotypes [36].

Genomic template stability (GTS) assessment is a measure of damage to DNA and DNA mutation (deletion or addition of DNA sequences, structural modification, point mutation, and mutation by polyploid variability). DNA bands from RAPD analysis can significantly contribute to molecular marker assemblies for the identification of damaged or mutant DNA in the cells of plants. GTS is a qualitative indication of variations in RAPD profiles. A study on *Gossypium hirsutum* L. (cotton) genotypes (Aleppo 118 abbreviated as A118, Deir-Ezzor 22 abbreviated as DE22, NIAB 78 abbreviated as N78, and Deltapine 50 abbreviated as DP50) was conducted to evaluate the response of genotypes cultivated in salt-affected fields (200 mM NaCl) and in a non-saline climate (control). Variations in RAPD profile were calculated by GTS as a percentage. Maximum values were observed in two sensitive genotypes, DP50 and A118, with GTS of 79.1% and 58.2%, respectively. In contrast, minimum values were observed in two salt-resistant varieties, DE22 and N78, that displayed 36.7% and 26.4% GTS, respectively. Changes in DNA might also be utilized for improving germplasm resistance to salinity stress in plant breeding strategies. Moreover, salt-resistant genotypes (DE22 and NB78) carrying the lowest values of GTS showed the highest polymorphic expression of RAPDs. Based on these results, RAPD analysis is useful for identifying DNA sequences linked to salt (NaCl) stress. Consequently, such DNA markers might provide breakthroughs in the preliminary detection of salt-resistant genotypes for cotton plants [37].

Wheat quality and production in many regions are substantially affected by drought. Losses due to drought are equal to losses due to other climatic factors. This condition is worsened by global climate change [38,39]. Drought-induced expression of various genes contributes directly to stress resistance [40]. Drought-resistant-related DNA primers were utilized in early DNA fingerprinting for RAPD analysis to examine genetic variation in wheat varieties. A similar study sought a genetic explanation for drought resistance in wheat hybrids. A RAPD primer P6 (TCGGCGGTTC) amplified a 920 base pair band in semi-drought-resistant and drought-resistant genotypes that were absent in drought-sensitive varieties. Further, a RAPD band of 717 base pairs (*Dreb-B1* gene) was located on the B genome derived from drought-resistant “Barakatli-95” [41]. Rashed et al. [42] found two negative and four positive RAPD markers that confirmed RAPD reliability for identifying drought-resistant wheat genotypes.

In horticultural plants, tomato crops are less productive in higher temperatures. Characteristics associated with yield are expected to be quantitatively inherited and primarily affected by the changes in environmental conditions. The complexity of characteristics makes crops difficult to assess, especially with respect to heat resistance. A study of RAPD molecular markers related to heat-resistant genes in tomatoes under high-temperature stress identified 43 recombinant inbred lines in F7 generations developed from a wild cross between heat-sensitive (L4422) and heat-resistant (CL 5915) parents. Among 200 RAPD markers, fourteen were identified as representing heat tolerance using bulked segregant analysis. A few RAPD molecular markers were specific to one character but most were associated with two. Heat resistance-related RAPD markers showed a positive gene impact due to the contribution of the CL5915 gene. Selection of genotype molecular markers specifically for heat tolerance can be enhanced by choosing individual genotypes with desired fruit trait markers (number, weight, and yield-related markers), such as P06, X01, D06, and D11 markers in tomatoes, which may help traditional breeding using CL5915 as a contributor parent [43]. Additional applications of DNA markers in horticultural crops with a focus on abiotic stress are listed in Table 1.

### 3.2. Stress Tolerance in Hybrids

**Figure 1:**
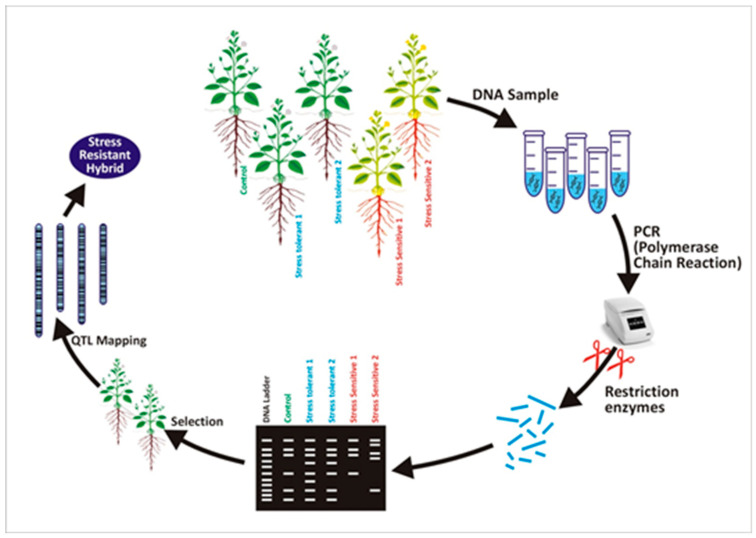
Mechanism of DNA marker-based progeny screening for stress-resistant characteristic.

Marker-assisted selection is an efficient tool for improving abiotic resistance in plants. Mechanisms of screening stress-tolerant hybrids by using DNA marker-assisted criteria are illustrated in Figure 1. Identification of stress-resistant genes commonly depends on SSR markers. SSR analysis, along with bulked segregant analysis, is useful for identifying molecular markers related to agronomic traits, such as grain filling and heat tolerance in wheat crops. Three markers (Xgwm132, Xgwm617, and Xgwm577) were identified by SSR analysis. These markers are linked with the rate of grain filling in hot environments. These approaches helped the development of a cultivar with improved resistance to heat stress. Moreover, SSR markers in rice, RM3586 (chromosome 3), and RM3735 (chromosome 4) expressed an effective link with heat resistance showing 3 and 17 percent total genetic variation, respectively [55,56,57].

SSR molecular markers assisted with identification of drought-resistant tetraploid cotton hybrids. Allelic polymorphic results using SSR primers and agricultural features showed significant findings in “Varamin” and “Sayar 314” hybrids, whereas the “Tabladila” hybrid showed highly polymorphic data with EST-SSR markers. The drought tolerant hybrid “Nazily” showed 53 percent polymorphism [58].

### 3.3. Genetic Diversity Identification under Heat and Frost Stress

Screening for heat-resistant varieties or genotypes under field conditions (morphological screening) is not preferable due to uncontrollable climatic influences that compromise a trial’s repeatability and precision. Moreover, guaranteed regularity of high temperature (heat stress) in growing areas is not possible. Genetic assessment of quantitative characteristics for adaptive responses is mandatory. Molecular analysis permits the utilization of specific genotypes in breeding strategies for enhancing yield stability and crop sustainability under severe stress [59].

Heat resistance is a multi-genetic characteristic with various components of resistance regulated by various sets of genes in different tissues or in different growth stages. Sequence-related amplified polymorphism markers (SRAP) are PCR type molecular markers that retrieve DNA fragments in a single PCR reaction. These DNA markers amplify many polymorphic and reproducible alleles and loci. SRAPs can amplify specific efficient and active genes, because gene sequences are necessary for the method. Due to their multiallelic and multilocus nature, these markers are often preferred for DNA fingerprinting, genetic diversity evaluation, and gene mapping. Random distribution across the plant genome is not suitable for the use of SRAP markers [60]. Another DNA marker, target region amplified polymorphism (TRAP), is an efficient and active PCR type marker that works with two 18-mer DNA primers. One primer is “fixed” from EST (expressed sequence tag), whereas the second primer is linked with either a GC- or AT-abundant core to pair with an exon or intron [61,62].

These markers were applied to wheat genotypes grown for heat resistance. A genetic analysis was carried out for SRAP and TRAP markers to evaluate genetic diversity in durum wheat genotypes. Genetic diversity in agronomic characteristics under heat stress was identified. Field performance data based upon agronomic traits used multi-genetic and complex forms. However, marker-assisted information of SRAP and TRAP analysis was valuable for identification of genetic diversity in an unbiased way compared to agricultural morphological evaluation [59].

In contrast, frost is a chief cause of reduced yield and death in pea crops. A field investigation to improve frost resistance of peas used 672 diverse pea genotypes at three different locations. Trait-based marker association was used to assess frost resistance with 267 SSR molecular markers. Among genotypes, 16 were detected as most winter-tolerant for their capability to live in all experimental fields in that study. Population structure showed a structured population of two sub-populations along with some combinations in the 672 genotypes. An association method identified seven molecular SSRs that continually showed a relationship with frost resistance in a minimum of two different environmental conditions with two statistical models. One marker is EST1109 on LG VI that was projected to localize with a gene that participates in glycoproteins metabolism in response to frost stress. This gene induces a different pathway for chill resistance in pea crops. These winter-resistant germplasms and cold-resistant linked markers play a role in marker-assisted breeding for cold-resistant cultivars of peas [63].

## 4. Mapping of QTL Genes Related to Abiotic Stresses by DNA Markers

### 4.1. Saltol

Salt-resistant quantitative trait loci (QTLs) have been identified and mapped to chromosomes of rice. One major mapped QTL is saltol, mapped to chromosome number 1 of the rice genome. After cloning of the saltol region, a gene was identified that was linked with the low uptake of Na^+^ and high absorption of K^+^ ions. This gene results in a low Na:K value phenotype under high salinity conditions [64].

For the mapping of genes, simple sequence repeat (microsatellite) markers are suitable because such DNA markers have been utilized to evaluate the genetic differences in many crops. Their reproducibility, simple protocol, and significant ability to discern polymorphisms and co-dominance are preferred for evaluating DNA profiles of plant germplasm. Progeny evaluation, evolutionary research, and mapping of genomes and genetic diversity is possible using these markers. A study addressed genetic diversity among rice genotypes using SSR markers present on rice chromosome number 1. These DNA segments are an important tool for assessing salinity resistance in rice seedlings. Research findings showed discrimination among SSR markers related to salinity-resistant haplotypes by referencing “Pokkali”, the main expressional source of salt-resistant QTL (saltol) on chromosome 1. QTL saltol was mapped on this chromosome utilizing eight generation inbred hybrids (Pokkali × IR29). Among 33 SSR markers, the RM8094 primer was revealed be significant in genetic differences. Cluster analysis distributed genotypes into three categories, which were comprised of 8, 12, and 16 genotypes. Highly salt-resistant IRRI selected breeds were clustered together into one class. Furthermore, the salt-resistant and moderately-resistant lines i.e. FL478 and Pokkali genotypes were clustered into the second group. Sensitive and highly susceptible lines were clustered separately into the third group. Expression of RM1 0745 and RM8094 DNA markers were valuable for discriminating salinity-resistant hybrids [65].

The saltol gene was transmitted into a popular cultivar of rice by marker-assisted breeding using genotypic and phenotypic screening. The SSR markers RM493 and RM3412b were effective in screening for the saltol gene. Saltol gene introgression into receiver cultivars can be achieved using co-dominant SSR markers. The donor parent FL478 was the main source for the successful transmission of the saltol gene (salt resistance) into the BT7 progeny genome [66].

### 4.2. Dehydrin

Dehydration stress induces significant molecular changes to decrease water loss. Responses of plant cells to dehydration stress involved an assembly of osmotically efficient components such as hydrophilic proteins and dehydrins. Plant stress resistance and production of dehydrin proteins or transcripts are positively interconnected in response to stress. Y_n_SK_m_ type hydrins are common in major agricultural crops, such as barley, which displays 10 of 13 dehydrin genes. Wheat also expresses Y_n_SK_m_ type hydrins. Dehydrins are actively synthesized in response to strong dehydration stress (frost, drought, and salt) and when abscisic acid is present in promoting regions [67].

Dehydrins are observed to act in a protective role during times of cellular dehydration by improving the activity and efficiency of enzymes when water is less available. Research using barley proved that two genes related to dehydrin play an important role in improving salt and drought resistance in wild and Tibetan barley. WRKY coded protein is controlled by the Hv-WRKY38 barley gene that is expressed mainly in response to drought and cold stress. These genes are genetically mapped near the QTL location [68,69]. Many genes are associated with osmotic stress, such as aquaporin and CBF, but the dehydrin response is very important in this context. Moreover, the CBF gene in plants actively participates in signaling pathways linked to salt and drought stress [70,71].

In *Citrullus colocynthis*, drought-tolerant genes were detected and sequenced utilizing a DNA marker analysis technique (ISSR). Four individuals from various locations were collected for genomic studies. Specific ISSR primers were developed related to drought-tolerant genes—UB, PEPKS, Dehydrin, ACT, and P5CS. These genes successfully identified drought resistance ability in individuals grown in four locations [72].

## 5. QTL Mapping by DNA Markers

Drought is a major abiotic stress that can damage productivity in different wheat-growing areas worldwide. DNA markers linked with QTLs specific to drought resistance might substantially improve drought tolerance in hybrids. A study on the detection of QTLs associated with grain yield genes under drought stress provided valuable genetic information. STS markers were used for QTL mapping for inbred wheat lines resistant to drought. QTL of grain yield is found on the proximal location of chromosome 4AL. This location is linked with the rate of grain filling, the density of spikes, grain yield, drought sensitivity index, and biomass production [39]. Stress-related QTLs in various agricultural crops and especially in wheat have been reported by various authors (Table 2 and Table 3).

Genetic diversity is high in rice due to progenitor species and wide distribution of plants across many hectares. Many stresses reduce yield and production. Up to 50% losses in yield are due to abiotic stresses alone. Salinity is a significant biophysical challenge for production in various rice-growing regions [85]. Resistance to salinity stress is complicated genetically and physiologically. Various stress-related QTL were identified in rice (Table 4).

Detection of QTLs for salinity resistance with closely associated contiguous DNA markers might be perfect for coping with conventional methods of breeding, which profoundly depends on morphological study [89]. In rice genotypes, twenty QTLs were detected on chromosomes 1, 2, 4, 6, 8, 9, and 12. Unique QTLs, i.e., qSESF12.1 and qSESI12.1, might be used to highlight mapping of the loci and to detect the closely connected contiguous markers for increasing the salt resistance [90].

Yield production and stability of *Pisum sativum*, commonly known as pea, is inhibited by drought in most climates. Less research is available on drought tolerance and associated genetic resources for pea. One study reported genomic loci linked to drought tolerance. Drought indications and relative water content of soil and in leaves during a period of drought stress were assessed for recombinant inbreed pea lines developed from two parents known to screen for drought resistance—ten quantitative trait loci linked with features independently describing from 9 to 33 percent of the morphological differences. Reproducible molecular markers were identified associated with QTLs. These molecular markers may be utilized to choose the individuals displaying required QTLs in pea breeding for drought tolerance [91].

Extending cold tolerance for production of cold season pea varieties is a significant challenge. Crossing of cold season cultivars needs to consider freezing resistance as well as seed yield and quality. Genetic determinants of cold/frost resistance and identification of genetic associations with developmental and yield features were the target of one study. A newly detected source of cold tolerance was used, and populations of recombinant inbred lines were assessed in six different climatic conditions. A genetic map consisting of 679 molecular markers across seven linkage groups and comprising 947.1 cM was generated. One hundred sixty-one QTLs accounting for 9 to 71 percent of morphological differences were identified for all measured traits. Findings showed that frost resistance may be bred individually to improve seed quality and productivity [92].

## 6. Marker-Assisted Selection by SNP Marker

Association mapping assesses more alleles in large populations than linkage analysis. Mapping shows the benefit of identifying evolutionary recombination and many various lines with mutational characteristics. Specifically, this method identifies genes associated with phenotypic diversity. Currently, the use of gene mapping analysis is important for recognition of genes responsible for quantitative variation of complicated features, e.g., resistance to drought [93,94]. Conversely, association mapping is weak for identifying rare alleles in plant populations. In addition, costs are higher due to the need for genotyping and sequencing abundant lines [94]. Utilization of fixed multiplex SNP chips is time-saving and cost-effective for association mapping and genome-wide linkage. In contrast, linkage analysis requires allele separation by capillary electrophoresis and multi-allelic markers. Studies confirm that SNP chips provide quality data, precision in genotyping information, and detailed genetic information. In addition, SNPs may be preferable for linkage analysis compared to traditional DNA markers, such as SSR. SNPs are advanced molecular markers, present in abundance with respect to variation. Functional genes and genetic differences are identified by SNP due to genome-wide assembly characteristics [95]. Hao et al. [96] identified 27 SNPs related to drought tolerance genetic variations in maize lines by detecting functional genetic differences.

## 7. Conclusions

Abiotic stress control in agricultural crops is mandatory for higher quality and yield. Molecular genetics provides numerous DNA markers that explore genetic modification, genotypic resistance, stress tolerant lines, and genetic information related to abiotic stresses. Early molecular marker technology provided DNA markers that offered basic information about stress resistance. However, current advanced marker applications can now identify specific genes or group of genes responsible for abiotic stress tolerance. Combining DNA markers with QTL mapping illustrates a pattern of stress tolerance genes on specific chromosomal loci. However, demand for continuous improvement in DNA marker technology will allow even more detailed analysis of stress tolerance as the climate changes.

## Figures and Tables

**Table 1:** Application of DNA markers in horticultural crops for abiotic stress.

Crop	DNA Marker	Abiotic Stress	Objective	Reference
*Petunia × atkinsiana*	RAPD	Salinity	Genetic diversity determination in mutant clones Screening of mutants related to salt resistance	[44]
D. Don cv. Prism Red.	ISSR			
Tall fescue (*Festuca arundinacea*)	Sequence characterized amplified region (SCAR)	Summer stress	Development of sequence-related markers to screen summer stress-resistant plants	[45]
Perennial grass (Miscanthus sinensis)	SSR	Drought	To formulate SSR markers linked to drought resistance by utilizing transcriptome sequencing	[46]
Salvia (*Salvia miltiorrhiza*)	AFLP	Drought	To segregate drought-related genes in sterile male and fertile near-isogenic lines of *S. Miltiorrhiza*	[47]
			To evaluate the change in fertility of plants during drought stress	
Strawberry *Fragaria ananassa* Duch.)	Expressed sequence tag (EST)	Drought	To assess the correlation between leaf WLR and RWC and specific DNA markers	[48]
	Amplified fragment length polymorphism (AFLP)		To test the utilization of association mapping in *Fragaria* genotypes to develop a group of correlated markers linked with physiological characters (participated in drought resistance)	
Citrus	Quantitative trait loci (QTL)	Salinity	Genetic evaluation of salt resistance concerning physiological and vegetative characteristics	[49]
*Citrus reshni*				
*Poncirus trifoliata*				
Tomato (*Solanum lycopersicum* L.)	SSR	Salinity	Molecular characterization concerning salt resistance characteristics mapping	[50]
			Development of an integrative multi-layer network connected to most selective SSR loci and genotypes to phenotypes recorded under salt stress	
Tomato (*Solanum lycopersicum* L.)	RAPD	Heat	To confirm all genotypes by SCAR that had been clearly recorded by RAPD markers	[51]
	Sequence characterized amplified region (SCAR)		SCAE-1 and SCAE-2 type of SCAR was identified to discriminate the tomato genotypes for heat resistance	
Tomato (*Solanum lycopersicum* L.)	Sequence-related amplified polymorphism (SRAP)	Heat	To investigate a genotypic arrangement of tomato genotypes with RAPD and SRAP molecular marker	[52]
	Randomly amplified polymorphic DNA (RAPD)		Genetic comparison and similarities of selected genotype with standard heat-resistant genotypes of tomato	
Tomato (*Solanum lycopersicum* L.)	Quantitative trait loci (QTL)	Heat	Detection of main heat-resistant QTLs in seedlings of tomato	[53]
			Identification of high-temperature stress-reactive genes within the main QTLs	
Cassava (*Manihot esculenta Cranz*)	Expressed sequence tags–simple sequence repeat (EST–SSR) markers	Drought	Marker-assisted selection of progeny tolerance to drought stress	[54]
			Identification of specific gene association related to drought stress resistance	

**Table 2:** Application of DNA markers for QTL mapping in agronomic crops.

Crop	Botanical Name	DNA Marker	No. of QTLs	No. of Chromosomes with QTLs Loci	Objective	Reference
Cotton	*Gossypium hirsutum*	Simple sequence repeats (SSR);			Salt resistance trait identification Mapping strength assessment for QTL detection	[73]
		Single strand conformation polymorphic (SSCP)	14	11	Inbred line developmental resistance to drought QTL detection linked to drought resistance	[74]
Maize	*Zea* *mays*	Single nucleotide polymorphism (SNP)	29	1, 3, and 5	Accessing salt resistance at the f seedling stage using unconditional and conditional QTLs	[75]
Sorghum	*Sorghum* *bicolor*	Restriction fragment length polymorphism (RFLP)	7	1 and 2	Drought resistance and lodging resistance detection before flowering	[76]
Barley	*Hordeum vulgare*	Single nucleotide polymorphism (SNP)	2 (salt tolerance indices)	1 and 2	To estimate the genetic difference of Asian barley for salt resistance To identify and screen out salt resistance traits in accessions	[77]
Barley		Simple sequence repeats (SSR)	13	4	Identification of 7 QTLs handling Na+, K+ content, and Na+:K+ ratio	[78]

**Table 3:** Application of DNA markers for QTL mapping in wheat (*Triticum aestivum*) crops.

DNA Markers	No. of QTLs Related to Tolerance	Chromosome No. with QTL Loci	Research Objectives	References
Simple sequence repeats (SSR) and amplified fragment length polymorphism (AFLP)	3	1, 5, 7	To classify and map QTLs for heat resistance To identify the DNA markers linked with QTLs	[79]
Simple sequence repeats (SSR), diversity array technology (DarT), gene-based marker for Vrn-A1	1	5A	Genetic structure of drought tolerance by reproductive stage Development of drought tolerance morphological method focusing on premature microspore stage of pollen development for eliminating stress at flowering time	[80]
Simple sequence repeats (SSR)	8	2A	To detect the linkages of SSR markers with drought resistance character on chromosome 2A	[81]
Single nucleotide polymorphism (SNP)	6	7A	High-density SNP association development in F2 population for salt tolerance QTLs and markers detection connected with different micronutrient concentrations and salt resistance relevant to seedlings	[82]
Single nucleotide polymorphism (SNP)	3	3B, 4A, and 5A	SNP identification related to heat tolerance	[83]
Simple sequence repeats (SSR)	2	8 and 10	Cold stress-related QTL detection for improving yield traits, except effects on spikelet fertility	[84]

**Table 4:** Application of DNA markers for QTL mapping in rice (*Oryza sativa*).

DNA Markers	No. of QTLs Related to Tolerance	No. of Chromosomes with QTLs Loci	Research Objectives	References
Simple sequence repeats (SSR)	47	1–4 and 6–12	Reliable QTLs for drought tolerance and yield performance under stress conditions Marker-assisted selection in rainfed areas	[86]
Single feature polymorphism, simple sequence repeats (SSR)	2	4 and 10	To detect possible QTL linked with high-temperature resistance Gene mapping of heat tolerance	[87]
Simple sequence repeats (SSR)	1	3	Heat tolerance related QTLS identification Marker identification for use in marker-assisted breeding	[88]
